# ZEB2 Gene Pathogenic Variants Across Protein-Coding Regions and Impact on Clinical Manifestations: A Review

**DOI:** 10.3390/ijms26031307

**Published:** 2025-02-03

**Authors:** Waheeda A. Hossain, Caroline St. Peter, Scott Lovell, Syed K. Rafi, Merlin G. Butler

**Affiliations:** 1 Departments of Psychiatry & Behavioral Sciences and Pediatrics, University of Kansas Medical Center, Kansas City, KS 66160, USA; whossain@kumc.edu (W.A.H.); c527s208@kumc.edu (C.S.P.); rafigene@yahoo.com (S.K.R.); 2 Protein Structure Laboratory, University of Kansas, Lawrence, KS 66047, USA; swlovell@ku.edu

**Keywords:** Mowat–Wilson syndrome, neurodevelopmental disorder, ZEB2 gene variants, protein domains and non-domain regions, multi-system organ development, genotype–phenotype relationships

## Abstract

Mowat–Wilson syndrome (MWS) is a rare multi-system genetic disorder caused by variants in the Zinc Finger E-Box-Binding Homeobox 2 (ZEB2) gene. ZEB2 is an autosomal dominant gene containing ten exons within the canonical version transcript (Isoform: O60315-1). The ZEB2 gene encodes six functional domains and seven non-domain regions. This review provides a comprehensive summary of pathogenic variants and their associated MWS clinical characteristics, focusing on ZEB2 pathogenic variants, functional protein domains and non-domain regions with clinical features. A systematic literature search from 2001 to 2023 and of unpublished datasets found 191 individuals with reported clinical features and genotypic data. Genetic defects and clinical manifestations were examined that presumably impact on the structure and function of the ZEB2 gene, thereby causing multiple developmental defects with corresponding clinical presentation. This study found more nonsense ZEB2 variants observed within exon 8, which encodes four of the six protein domains: the CtBP-interacting domain (CID), homeodomain (HD), SMAD-binding domain (SMD or SBD) and part of the N-terminal zinc finger cluster (N-ZF), suggesting exon 8 plays a crucial role in this protein structure and function with multi-organ involvement. Exon 8 defects were found to be statistically more represented for gastrointestinal findings when compared to other exons, while frameshift defects were more often seen for the typical MWS face in non-domain protein regions. In contrast, nonsense or other types of variants in exons 3, 4 and 5 which encode only flanking non-domain regions were observed more often, compared with other exons excluding exon 8, to be specifically involved in the MWS facial gestalt, brain malformations, developmental delay and intellectual disability. Deleterious ZEB2 frameshift (45%) and nonsense (38%) gene variants were most often observed with deletions at 6% and missense at 5%. The genotype and clinical relationships in MWS can provide insights into prognosis, morbidity, clinical surveillance strategies and counseling of family members.

## 1. Introduction

Mowat–Wilson syndrome (MWS-OMIM# 235730) is a rare congenital genetic disorder first described in 1998 [1]. The prevalence of the syndrome is estimated at one per 50,000 to 70,000 (OMIM-Online Inheritance in Man: https://www.omim.org/, accessed on 7 January 2025). It is identified with consistent phenotypical features across ethnic groups. The characteristic facial gestalt of MWS includes a high forehead, frontal bossing, large eyebrows with medial flair, hypertelorism, deep-set large appearing eyes, large and uplifted ear lobes with a central depression, a saddle nose with a prominent round nasal tip and columella, an open mouth with an M-shaped upper lip, frequent smiling and a prominent but narrow triangular pointed chin. Developmental delay is common, while other phenotypical features are more variable [2,3,4,5,6].

The diagnosis of MWS is primarily based on clinical features, with confirmation by genetic testing. The hallmark features include distinct dysmorphic facial features, developmental delay/intellectual disability and a pathogenic heterozygous variant in the ZEB2 gene or a full/partial deletion of chromosome 2q22.3, including the ZEB2 gene. Molecular testing allows for definitive diagnosis and enables targeted genetic testing in individuals suspected of having MWS. Most pathogenic variants of the ZEB2 gene are de novo and associated with loss-of-function (nonsense, frameshift, splicing mutations and deletions) or with missense gene variants which may show less clinical severity [7]. A review of ZEB2 gene variants was summarized from the literature by St. Peter et al. [8], including in-frame defects with intragenic deletions, partial duplications, or insertion deletions along with a case report. They also reviewed ZEB2 gene variants, types and frequencies, protein modeling, defects and molecular interactions impacting multi-system embryonic development.

There are six protein domain regions encoded by the ZEB2 gene, along with non-domain or linker protein regions. In addition, other ZEB2 gene regions include introns, untranslated regions (UTRs), promoters, enhancers and long non-coding RNAs (lncRNAs) that play a role in gene regulation and protein function. Non-coding regions are key to modulating gene expressions [9].

This review aims to assess the current molecular understanding and causes of MWS, focusing on the relationship between ZEB2 gene variants and clinical phenotypes, including multi-organ involvement. Specifically, we evaluated the types and locations of gene variants impacting both protein domain and non-domain regions with an influence on protein production and clinical manifestations. Additionally, we sought to identify gaps in current knowledge to enhance the phenotypic identification of MWS based on gene variants and protein production, ultimately encouraging further research on this rare genetic disorder.

## 2. Subjects and Methods

### 2.1. Literature Search

A recent literature review of PUBMED and unpublished databases, as reported by St. Peter et al. [8], served as the basis for our analysis of the genetic and clinical data related to Mowat–Wilson syndrome. They conducted a comprehensive search of published literature from 2001 to 2023 and previously unreported patient databases, focusing on keywords such as “Mowat–Wilson syndrome”, “ZEB2 gene” and “protein defects/variants”. This research utilized the PUBMED database (accessed on 1 October 2023), focused on human studies. and initially identified approximately 180 published articles. After applying inclusion and exclusion criteria for accuracy, 20 publications had sufficiently detailed human genetic and clinical data to be analyzed. A total of 298 individuals with MWS were identified, including in unpublished data from the MWS Foundation (www.mowat-wilson.org), and reported by St. Peter et al. [8]. The authors summarized reported ZEB2 gene variants, their types and frequencies and their corresponding encoded protein defects impacting structure and domains, with more than 90% of the gene defects classified as nonsense or frameshift changes. They further explored and described other genes predicted to interact with the ZEB2 gene, their gene–gene molecular and encoded protein functions, and the biological networks and protein-binding effects on embryonic development, including craniofacial, spine, brain, gastrointestinal, kidney, cardiovascular and hematopoiesis effects. We reanalyzed data from this original report to meet the goal of our current study and focused specifically on 191 individuals with MWS who had sufficient clinical, phenotypic and genetic data. The reported or published MWS cohorts consisted of individuals from Japan (n = 63), China (n = 49), Poland (n = 29), Australia (n = 3) and one each from Indonesia, Egypt, Ireland, Colombia and Turkey, with the remaining from the USA. Individuals lacking comprehensive information on their phenotype and/or development were excluded. The published data from each source informed our review and became the basis for our genotype–phenotype correlation study. Chi-square tests with Yates correction were used for statistical analysis.

We also utilized the STRING database (https://string-db.org) to further identify predicted protein–protein associations, functional interactions and biological networks to identify the top ten biological processes, molecular functions, cellular components, pathways and disease–gene associations [10] for the ZEB2 gene. Other computer-based searchable websites geared to search for and analyze human disorders, genes, and protein structures and functions were used. These include the Online Mendelian Inheritance in Man (OMIM) (www.omim.org), which is a catalog of human genetic disorders and genes, UniProt (www.uniport.org), which is a free searchable database of protein sequences and functional information with the capability to search, align, map, annotate and download proteins, with entries derived from genome sequencing projects, and Ensembl (www.ensembl.org/), which is a genome browser for vertebrate genomes that supports research in comparative genomics, evolution, sequence variation and transcriptional regulation.

### 2.2. Demographics

A total of 191 individuals with MWS were included, with gender reported in 188 patients: 52% were females and 48% were males. Age was reported for 157 individuals, with an approximate average age of 9.1 years (median = 6 years; range 0–45 years). An additional 28 individuals had age ranges from 3 months to 28 years but were grouped, as no individual age data were available.

## 3. Results and Discussion

### 3.1. ZEB2 Gene and Protein Description

#### ZEB2 Gene

The ZEB2 (Zinc Finger E-box-Binding Homeobox) gene encodes a member of a family of proteins consisting of transcription factors required for key cellular processes such as cell differentiation, development and epithelial–mesenchymal transition (EMT) for embryo formation [11]. The ZEB1 and ZEB2 genes have close homology and reflects a strong binding affinity for E-box DNA sequences [12] and are two of the most well-known members of this family. They both show an interplay between EMT transcription factors regulating hematopoietic stem and progenitor cell differentiation and hematopoetic lineage [13]. In addition, ZEB transcription factors are involved with miR200, a member of the microRNA family required to stabilize epithelial phenotypes [14].

**Figure 1:**
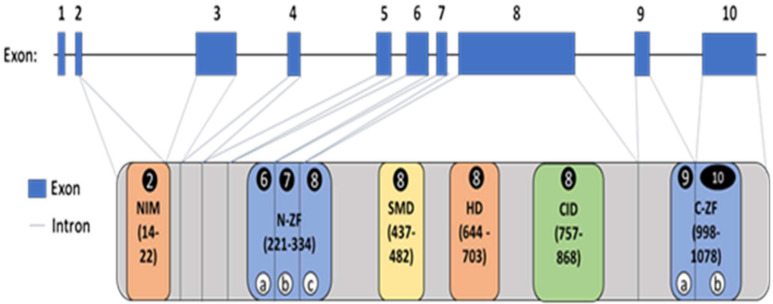
Representation of ZEB2 gene exons and introns with encoded protein structure with six domains having known protein functions and multiple non-domain regions of linker polypeptides. Image provided and modified from St. Peter et al. [8] with permission.

The ZEB2 gene is composed of 10 exons and encodes six protein domains (NIM, N-ZF, SMD or SBD, HD, CID and C-ZF) involved in folding and carrying out functions of the ZEB2 protein (see Figure 1). These six domains account for 55% of ZEB2 gene transcription as represented in Figure 1, while several under-characterized non-domain (linker) polypeptide regions comprise the remaining 45%, facilitating the formation of transcriptional complexes via protein–protein interactions. Exon 8 comprises 54% (or 656 amino acids—AA) of the total DNA base pairs coding for the ZEB2 protein, making it the largest exon. The other smaller exons are exon 2 (2% or 24-AA), exon 3 (7% or 87-AA), exon 4 (2% or 24-AA), exon 5 (5% or 63-AA), exon 6 (6% or 71-AA), exon 7 (3% or 37-AA), exon 9 (5% or 61-AA) and exon 10 (16% or 191-AA) (https://useast.ensembl.org). Exon 1 does not encode proteins (www.uniprot.org).

Given the size discrepancy, exon 8 encompasses a significant portion of the ZEB2 protein’s functional domains, including one-third of the N-ZF, the entire inter- or non-domain region between the N-ZF and SMD, the CID domain and half of the interdomain region between CID and C-ZF domains. Therefore, it is intuitive that exon 8 encodes more functional protein domains and more frequently harbors MWS phenotype-causing mutations. Early studies on MWS have shown that mutations in the ZFHX18 gene also known as Smad-interacting protein-1, SIP1, and now ZEB2, have demonstrated possible mechanistic insights for aberrant functions of single-protein domains of the multi-domain ZFHX1B/SIP1/ZEB2 protein [15]. However, when considering exon size, smaller exons such as exons 3, 6 and 7 for ZEB2 appear to show a higher per-nucleotide relationship with specific phenotypic features, especially with the typical MWS face.

ZEB2 gene expression is controlled in a tissue- and time-specific manner. It is composed of non-coding regions, which include introns, untranslated regions (UTRs), promoters, enhancers and long non-coding RNAs (lncRNAs), all contributing to gene regulation and protein functions (www.uniprot.org; www.genecards.org). Non-coding regions like introns and UTRs are known to affect mRNA stability, localization and translational efficiency. In ZEB genes, introns may harbor enhancer elements that play roles in tissue-specific expression, including in brain regions [16], while promoters and enhancers are found in non-coding regions crucial for controlling the spatial and temporal expression of genes. Non-coding regions can also recruit transcription factors that directly regulate ZEB gene expression and long non-coding RNAs may interact with ZEB genes to regulate their transcriptional activity, often impacting pathways such as epithelial–mesenchymal transition [17,18]. However, early research has documented Zeb2 mRNA expression in post-implementation amphibian and mouse embryos involving the neural epithelium and formation of the neural plate and the developing brain cortex [19].

In the context of Mowat–Wilson syndrome, the regulation of the ZEB2 gene by non-coding regions is especially critical, as mutations or disruptions in these regions may influence the phenotypic variability associated with the disorder. Specific intronic mutations within ZEB2 have been linked to splicing anomalies that lead to either truncated or functionally deficient ZEB2 protein products. Additionally, non-coding variants in the 3′ UTR region of ZEB2 can affect microRNA (miRNA) binding, particularly miR-200 family members, which have been shown to regulate EMT pathways vital to neural crest development, an area heavily impacted in MWS [20], which is supported by earlier studies in mice leading to a wide range of neurcristopathies [21]. Disruptions in miRNA interactions or enhancer activity may contribute to neural crest cell migration abnormalities, one of the hallmark features of MWS. Furthermore, lncRNAs such as ZEB2-AS1 have been shown to modulate ZEB2 expression through chromatin remodeling and any disruptions to this regulatory mechanism can affect not only neural development but also gastrointestinal and craniofacial features, common in MWS patients. These findings further suggest that non-domain regions may play a role in modulating the expression of ZEB2, contributing to the broad phenotypic spectrum observed in MWS.

Another genetic factor that may play a role in ZEB2 function is small nucleolar RNA (snoRNA), a class of small RNA molecules that primarily guide chemical modifications of other RNAs, such as ribosomal RNAs (rRNAs), transfer RNAs (tRNAs) and small nuclear RNAs (snRNAs). These are predominantly involved in the methylation and pseudouridylation of RNA, contributing to the maturation and proper function of rRNAs. SnoRNAs are typically found in the nucleolus of eukaryotic cells and classified into two main families based on their structure and function. SnoRNAs have been linked to t regulation of pre-mRNA splicing [10]. Like many other genes, the ZEB2 gene undergoes alternative splicing, particularly in untranslated regions. Regulatory snoRNAs may influence the splicing process of ZEB2 transcripts by guiding modifications of RNA processing machinery or participating in the control of splice site selection. Though direct connections between ZEB2 and specific snoRNAs are not well characterized, there are cases whereby snoRNAs play regulatory roles in other genes with similar regulatory pathways. For instance, SNORD116 is implicated in Prader–Willi syndrome and has downstream effects on gene regulation, playing crucial roles in pathogenesis [22]. A similar snoRNA could potentially influence ZEB2 expression, especially given its involvement in neurodevelopment and epithelial–mesenchymal transition processes, which snoRNAs might modulate through indirect mechanisms. Hence, snoRNAs may have broader implications for gene regulation, including for ZEB2, through complex RNA-based mechanisms [23].

### 3.2. ZEB2 Protein Domains and Non-Domains

Various genes encode a polypeptide comprised of multiple independent protein domain regions that fold and function independently [24]. These multi-domain proteins often contain several hundred to more than a thousand amino acids in which the functional domains are connected by linker (non-domain) polypeptides that are highly flexible [25,26]. Domains found in various proteins may provide catalytic, transmembrane or binding capabilities, but several domains may appear in different proteins. Protein domains are globular regions usually ranging in size from 50 to 350 amino acids in length, while shorter domains such as zinc fingers, are typically stabilized by cysteine and/or histine residues. Protein domains can fold and often form functional units [27,28,29,30,31]. They are independently stable and can be exchanged with other proteins to make chimeric proteins. These serve as modules for building large assemblies such as muscle fibers or can provide specific catalytic or binding sites needed in regulatory proteins or enzymes [32].

Six protein domains make up the ZEB2 protein. The NIM (nucleosome remodeling and deacetylase interaction motif) protein domain interacts with the NuRD complex (e.g., 15), which in turn facilitates major chromatin remodeling. The N-ZF (N-terminal zinc finger cluster) and C-ZF (C-terminal zinc finger cluster) domains play significant roles in gene regulation and function [33]. including early neural induction, as seen in Xenopus [34]. Both zinc finger clusters (N-ZF and C-ZF) are also essential for ZEB2 and the DNA-binding ability required to pack and modify DNA and to regulate gene expression crucial for proper functioning of DNA binding in vitro [35].

The SMD (SMAD-binding transcription factor domain) protein domain is critical for cell growth and development and mediates growth factor responses to TGF-beta signaling and/or BMP transcription during metazoan embryo development and for the regeneration of adult tissue homeostasis [13,36,37]. The HD (homeodomain) protein domain also regulates the expression of other genes during development [38,39]. The CID (CtBP-interacting domain) protein domain functions to regulate transcription and, as a corepressor in the nucleus [40,41,42], enhances transcription and translation, with an impact on neural crest cells [43]. The CtBP-interacting domain (CID) is responsible in vertebrates, particularly in neuronal cells, for the interaction of ZEB2 with CtBPs found in repeated PLDLS-like motifs, making ZEB2 more efficient at transcriptional suppression. CtBPs cannot bind DNA in a gene/promoter-specific context, but rely on the recruitment of DNA-binding transcription factors, such as ZEB2, to function [17] and when disturbed could lead to neuronal defects and single-cell lamination problems [41,42].

In our study, the largest number of organ systems affected may involve the HD domain and followed by SMD and CID. For example, SMD plays an important role in vertebrate embryogenesis, involving several tissues and differentiating cell types. This domain interacts with several proteins, including TGF-beta. These three protein domains are encoded by exon 8 (see Figure 1), which accounts for approximately 60% of the total ZEB2 protein. This protein is a member of the Zfh1 family of two-handed zinc finger/homeodomain proteins located in the nucleus and functions as a DNA-binding transcriptional repressor that interacts with activated SMADs. The protein isoform used in this study has 1214 amino acids and is designated as UniProt: O60315-1, a protein sequence and function database for the study of proteomics (www.uniprot.org). The gene–gene or protein interactions and binding involved with ZEB2 were described by St. Peter et al. [8]. In addition, non-domain regions in proteins are found between domains and constitute flexible linkers consisting of short relatively unstructured lengths of polypeptide chains. They may play a role in the assembly of macromolecular arrays and binding abilities as well as post-translational modifications. The relationships between proteins cannot be determined without examining their three-dimensional structures [32,39,40].

### 3.3. ZEB2 Gene Variants and Organ System Involvement

MWS is an autosomal dominant complex disorder with a spectrum of organ system involvement including cardiac, craniofacial and brain malformations with reported functional null ZEB2 mutations and includes nonsense with frameshifts reflecting the influence of ZEB2 gene on neural crest cells and neural tube development [44]. Microcephaly is also seen in more than 80% of MWS cases [3], consistent with the role of ZEB2 transcription factor in regulating intracranial and cortical–subcortical connections [45], including corpus callosum abnormalities in about 75% of MWS patients [46]. Epilepsy is also a major feature with causative brain malformations seen in MWS [6,47,48,49]. GABAergic disturbances may also play a functional role, with an imbalance in levels impacting cortical/subcortical interneurons and synaptic errors, leading to EEG discharges and seizures [48].

ZEB2 also plays a role in the development of the peripheral nervous system, including enteric nerves for the gut and for sensory neurons, leading to the under-reaction to pain frequently observed in MWS and in Hirschsprung disease. Moreover, constipation without Hirschsprung disease is present in additional patients [1,50]. In 2001, Amiel et al. [51] reported large-scale deletions in the 2q22 chromosome region containing the ZEB2 gene and truncating mutations in syndromic Hirschsprung disease (HSCR), with the involvement of midline structures, a common malformation of neural crest-derived enteric neurons and frequently associated with other congenital abnormalities. They screened 19 patients with HSCR and other clinical features and found large-scale deletions or truncating mutations in 8 of 19 patients with MWS. An early report showed three of four cases with MWS and HSCR had mutations located in exon 8 of the ZEB2 gene [52].

To address the question of a relationship between clinical and gene defects in MWS, Zweier et al. [53] classified 70 patients as having typical, ambiguous or atypical MWS according to their facial phenotype and clinical presentation. They were studied with FISH, quantitative PCR and DNA sequencing and deletions, splice sites or truncating mutations were detected in all 28 patients classified as having typical MWS. No gene defect was found in the remaining 15 cases with ambiguous facial features or in the 27 atypical patients. A lack of missense mutations in MWS and MWS-like patients was noted and further supported by Ghoumid et al.’s finding [54] that missense mutations lead to a mild MWS phenotype. A genotype–phenotype analysis proposed that deletions and frameshift or stop gene mutations resulted in recognizable facial dysmorphism with severe learning problems and variable malformations such as HSCR and heart defects, requiring more studies. Other studies have reported over 100 mutations of the ZEB2 gene in clinically typical patients with MWS and nearly all showed whole gene deletions or truncating mutations (nonsense or frameshift), further suggesting that haploinsufficiency is the basis of MWS pathology [55].

All 191 patients with MWS in our study showed pathogenic variants in the ZEB2 gene. The most prevalent variant type was frameshift (n = 86, 45%), with 34% found in protein domains with known functions and 66% found in non-domain regions located between domains. Among the 86 subjects, the frameshift variants included frameshift alone (n = 78) and frameshift plus other variants such as frameshift with small deletion (n = 5), a frameshift with small indel (n = 1), a frameshift with small insertion (n = 1) and a frameshift with a missense variant (n = 1). Other variants included nonsense (n = 72, 38%), with 56% found in protein domains and 44% in non-domain regions. Large deletions (n = 8, 4%) included four with large deletions and four with chromosome deletions, all found in non-domain regions. Deletions (n = 11, 6%) included four whole gene deletions and seven deletions in non-domain regions. Missense variants (n = 10, 5%) were found with 80% in protein domains and 20% in non-domain regions. There were two splicing mutations, an insertion deletion and a partial duplication (n = 4, 2%), and all were found in protein domains, except for a partial duplication that was found in a non-domain region.

MWS is a condition with a wide range of clinical findings ranging from mild to severe, including for neuronal development. Cordelli et al. [7] raised a possible association between ZEB2 gene defects and neurological phenotypes by errors in the formation and regulation of neural crest cell differentiation and migration as well as the modulation of GABAergic transmission. These errors are implicated in brain and head malformations, epilepsy, and sleep disorders with enteric and peripheral nervous system formation with cognitive and behavioral problems. For example, Ivanovski et al. [6] reviewed 87 patients with MWS and deletions that encompassed the whole ZEB2 gene and found more severe clinical manifestations such as more neurodevelopmental delay and problems. Two patients with intragenic variants without typical haploinsufficiency effect showed a mild phenotype with no epilepsy and mild to moderate intellectual disability. No patients with missense mutations were found in their study. Other studies have reported patients with ZEB2 missense mutations with a milder neurological and electroclinical phenotype [54,56].

**Figure 2:**
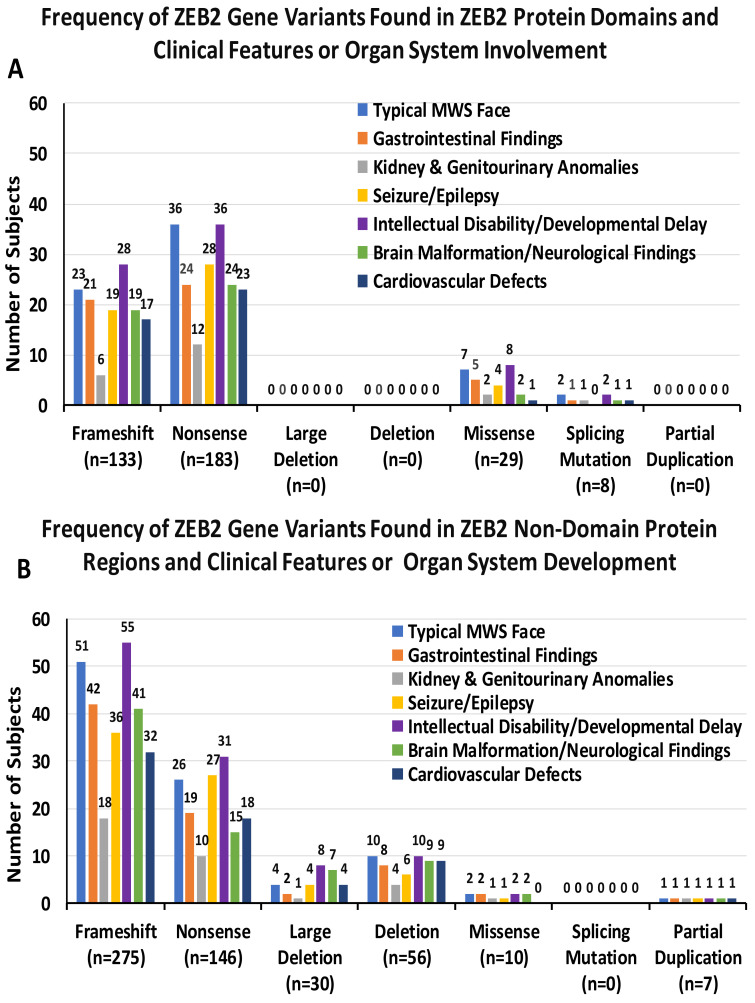
Frequency of ZEB2 gene variants compared with clinical features and organ systems or functions in 191 reported individuals with MWS; a specific gene variant may involve more than one clinical feature or organ system. (**A**) Frequency of ZEB2 gene variants located in protein domains and clinical features or organ system involvement. (**B**) Frequency of ZEB2 gene variants located in protein non-domain regions and clinical features or organ system involvement. Data utilized from literature sources [8,54,57,58,59,60,61,62,63,64,65,66,67,68,69,70,71,72,73].

The frequency of ZEB2 gene variant types and clinical phenotype/organ system involvement were analyzed across multiple organ systems in our study. Frameshift defects were followed by nonsense variants as the most prevalent. Frameshift mutations were most often associated with the typical MWS facial phenotype (46%, 14% found in protein domains and 32% in non-domain regions), while nonsense variants accounted for 38% (22% in protein domains and 16% in non-domain regions). Gastrointestinal findings showed 50% frameshift variants (17%) found in protein domains and 33% in non-domain regions) and 34% were nonsense (19% in protein domains and 15% in non-domain regions). Kidney and genitourinary anomalies were found with 37% frameshift (9% in protein domains and 28% in non-domain regions), while 34% showed nonsense (19% in protein domains and 15% in non-domain regions). Seizure/epilepsy had 43% frameshift variants (15% in protein domains and 28% in non-domain regions), while 43% showed nonsense (22% in protein domains and 21% in non-domain regions). Intellectual disability/developmental delay showed 46% frameshift variants (15% in protein domains and 31% in non-domain regions) and 37% showed nonsense (20% in protein domains and 17% in non-domain regions). Conversely, brain malformations showed 50% frameshift variants (16% in protein domains and 34% in non-domain regions), while 32% were nonsense variants (20% in protein domains and 12% in non-domain regions). Additionally, cardiovascular defects were observed in 46% of frameshift variants (16% in protein domains and 30% in non-domain regions) and 38% showed nonsense (21% in protein domains and 17% in non-domain regions). Deleterious frameshift and nonsense gene variants were more often observed when compared to missense or deletions, indicating the importance of these deleterious defects playing a critical role in MWS, particularly in non-domain protein regions. The smaller numbers found for deletions may represent that large deletions may include other genetic information along with ZEB2, potentially leading to spontaneous abortions. No deletions were found in the reported patients with MWS involving the domain regions for cardiovascular defects but were found in the non-domain regions (see Table 1 and Figure 2A,B).

### 3.4. Clinical Manifestations and Organ System Development

#### 3.4.1. Typical MWS Face Description

**Figure 3:**
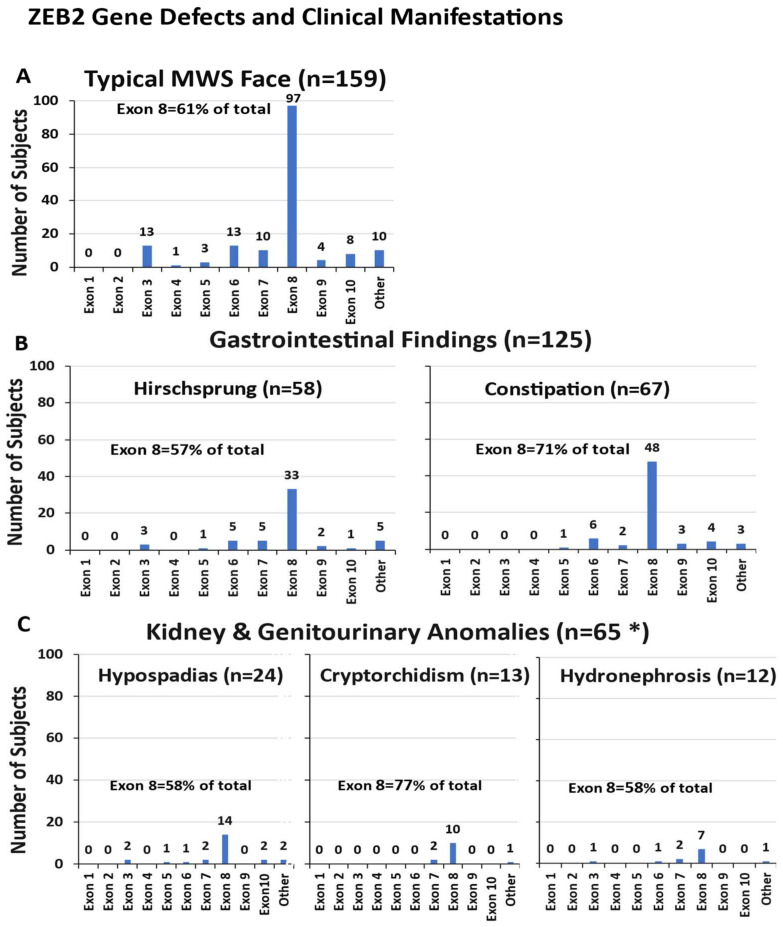
ZEB2 gene exon frequencies with associated clinical features and organ system involvement in individuals with MWS. (**A**) Typical MWS facial features. (**B**) Gastrointestinal disturbances. (**C**) Kidney and genitourinary anomalies. * Only subjects with single defects are shown in the histogram. Some have more than one defect while “Other” had more than one exon involved. Data utilized from literature sources [8,54,57,58,59,60,61,62,63,64,65,66,67,68,69,70,71,72,73].

Among the 191 MWS patients in our review, 83% (159 cases) had a distinctive facies with a high forehead, frontal bossing, large eyebrows with medial flaring, hypertelorism, prominent, deep-set eyes, uplifted ear lobes with central depression, a protruding nasal tip, and an open mouth with a prominent, narrow triangular pointed chin. Exon 8 was the most frequently observed exon involved with the characteristic MWS facial features, followed by the smaller exons 3, 6 and 7 (see Figure 3A).

#### 3.4.2. Gastrointestinal Involvement

Gastrointestinal (GI) disturbances, especially Hirschsprung disease, have been widely associated with MWS and found in nearly half of reported patients [5]. In our study, 65% (125 out of 191 cases) were identified with either Hirschsprung disease (n = 58) or more non-specific GI findings or dysfunction such as constipation (n = 67). As depicted in Figure 3B, exon 8 defects were found in 57% of cases with Hirschsprung disease and 72% of cases had constipation, with exon 6 being the second most commonly involved exon in both conditions.

#### 3.4.3. Kidney and Genitourinary Anomalies

Of the 191 reported patients with MWS, 65 cases, or 34%, had some form of kidney and urinary system abnormality or genital defects. Kidney and urinary system findings included hydronephrosis, pelviectasis, bilateral megaureters, a duplicated renal pelvis, a posterior ureteral valve, renal hypoplasia, lower pole lobulation, megaureter, neurogenic bladder kidney reflux, pelvis dilatation, vesicoureteral reflux, a cystic kidney or urolithiasis. Genital findings included an anteriorly placed anus, a bifid scrotum, cryptorchidism, micropenis, hypospadias and a webbed penis. In our study, 28 individuals (43%) had kidney and urinary system problems, while 37 individuals (57%) had genital involvement. As shown in Figure 3C, exon 8 was most often disturbed.

#### 3.4.4. Developmental Delays and Intellectual Disabilities

**Figure 4:**
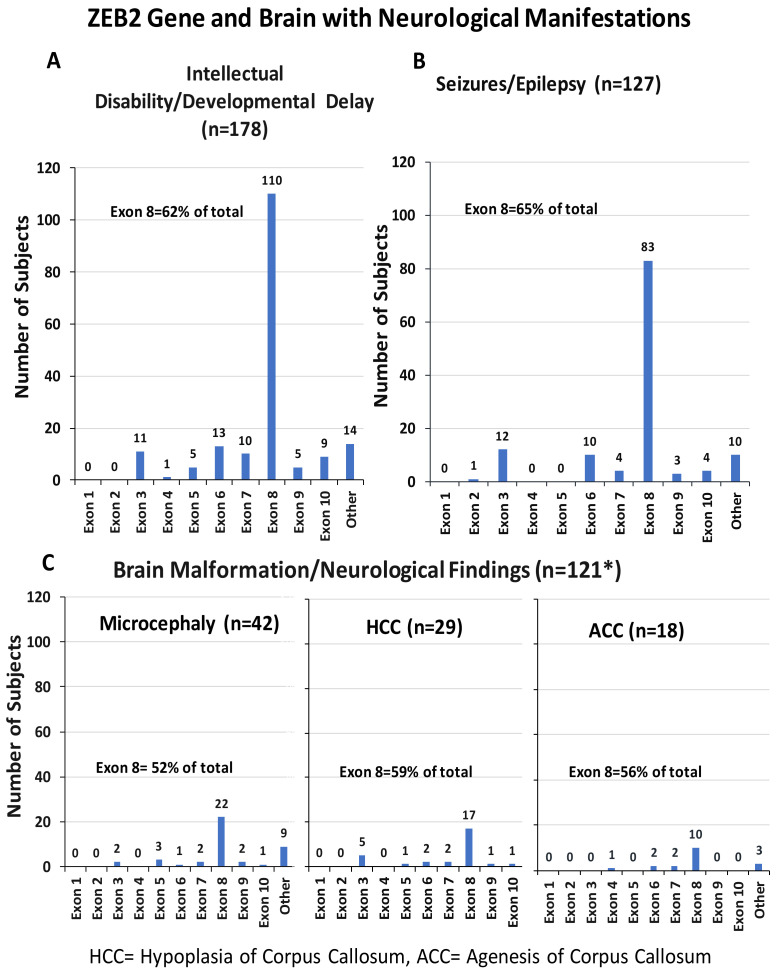
ZEB2 gene exon frequencies with associated clinical features and organ system involvement in individuals with MWS. (**A**) Intellectual disabilities and developmental delay. (**B**) Seizures and epilepsy. (**C**) Neurological manifestation. * Only subjects with single defects are shown in the histogram. Some have more than one defect while “Other” had more than one exon involved. Data utilized from literature sources [8,54,57,58,59,60,61,62,63,64,65,66,67,68,69,70,71,72,73].

Of the 191 reported patients with MWS, 178 (93%) had documented developmental delays or intellectual disabilities in older patients. In most cases they were described as severe, but a few were listed as moderate or mild. Exon 8 was involved in 62% of cases, followed by exons 6 and 7 (see Figure 4A).

#### 3.4.5. Description of Neurologic Manifestations and Seizures

Of the 191 individuals, 152 showed brain and neurologic manifestations, of which 121 (80%) had brain malformations. Neurological defects included microcephaly, agenesis of corpus callosum (ACC), corpus callosum hypoplasia, colpocephaly, callous body dysgenesis, lateral and third ventricular dilatation, cortical atrophy, incomplete hippocampal inversion, enlargement of the thalamus, caudate nucleus, myelin dysplasia, cortical atrophy or ventriculomegaly. Several patients had a single brain abnormality, such as microcephaly (n = 42), hypoplasia/aplasia of the corpus callosum (HCC) (n = 29) or agenesis of the corpus callosum (ACC) (n = 18), while others had a combination of findings, including microcephaly + HCC (n = 12) or microcephaly + ACC (n = 3). Exon 8 defects accounted for more than 50% of cases, as seen in Figure 4C. Seizure/epilepsy data were reported as Yes = 127, No = 52 and No available data = 11. Exon 8 defects were found for 65% of cases followed by exons 6 and 7 (see Figure 4B).

#### 3.4.6. Cardiovascular Defects

**Figure 5:**
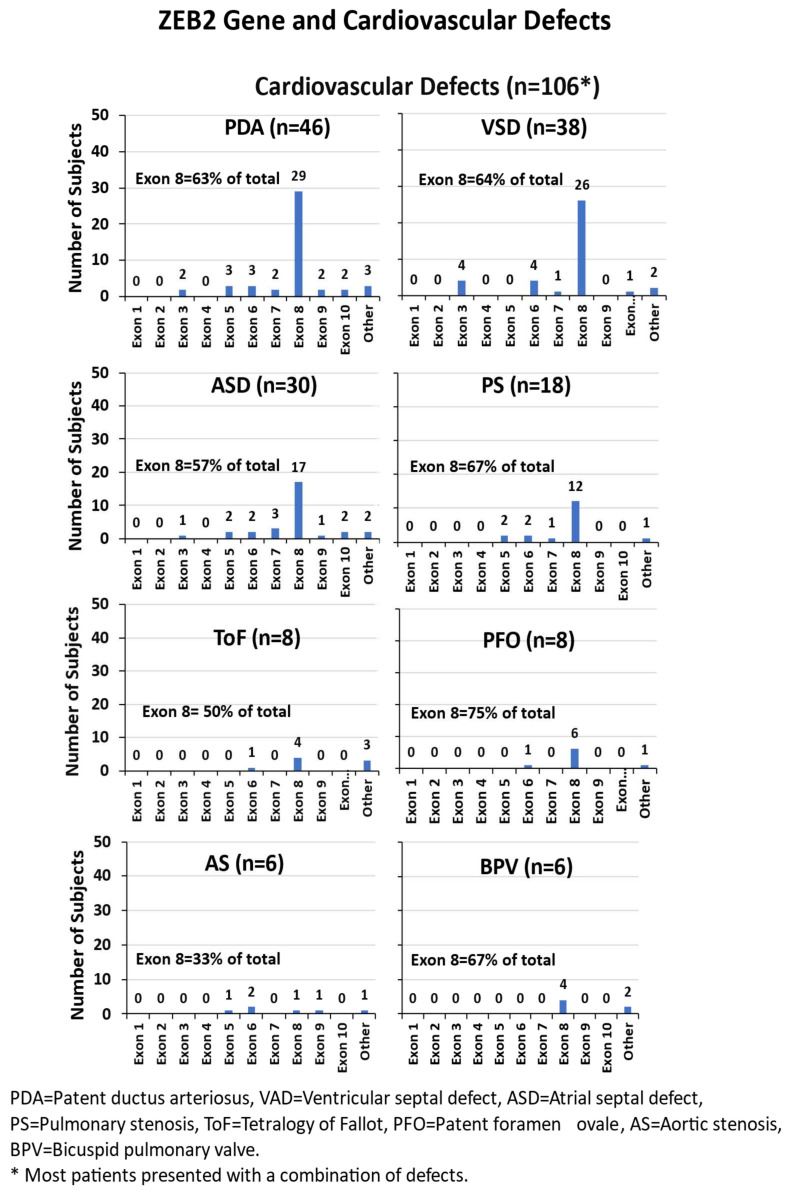
ZEB2 gene exon frequencies of various cardiovascular defects are shown in individuals with MWS. * Only subjects with single defects are shown in the histogram. Some have more than one defect, while “Other” had more than one exon involved. Data utilized from literature sources [8,54,57,58,59,60,61,62,63,64,65,66,67,68,69,70,71,72,73].

Congenital heart defects were seen in 106 (55%) of the 191 individuals with MWS, including a wide range of findings such as patent ductus arteriosus (PDA), ventricular septal defect (VSD), atrial septal defect (ASD), pulmonary stenosis (PS), tetralogy of Fallot (ToF), patent foramen ovale (PFO), aortic stenosis (AS), pulmonary artery sling (PAS), pulmonary atresia (PA), aortic coarctation (AC), bicuspid pulmonary valve (BPV), bicuspid aortic valve (BAV), Ebstein anomaly, pulmonary hypertension (PH), double outlet right ventricle (DORV), mitral regurgitation (MR), pulmonary regurgitation (PR), tricuspid regurgitation (TR), left superior vena cava (LSVC) and atrioventricular septal defect (AVSD). Another rare heart defect was found, referred to as Shone’s complex, which affects blood flow to and from the left side of the heart. Among these cardiovascular anomalies, exon 8 defects accounted for more than 50% of the cases, except for aortic stenosis, where the number of cases was notably low (n = 6) (see Figure 5).

#### 3.4.7. Other Phenotypic Findings

An additional 24% (45 of 191) of individuals with MWS exhibited various phenotypic defects that did not fit into a predefined category, such as eye problems. These included microphthalmia, blepharoptosis, astigmatism, vertical nystagmus, squint, bilateral cataracts or iris colobomas, exotropia, convergent strabismus, esotropia, exotropia, hypermetropia, myopia, pale optic discs or right ptosis. Hearing abnormalities were also noted and included external ear abnormalities, bilateral sensorineural hearing loss, conduction deafness or otitis media. Other reported abnormalities included craniosynostosis, hypoplasia sacral dimple, submucosal cleft palate, spina bifida, hypothyroidism, kyphosis, inverted duodenum, liver hemangioma, spleen hypoplasia or self-injurious behaviors.

#### 3.4.8. ZEB2 Exons and ZEB2 Protein Domains and Non-Domain Regions

**Figure 6:**
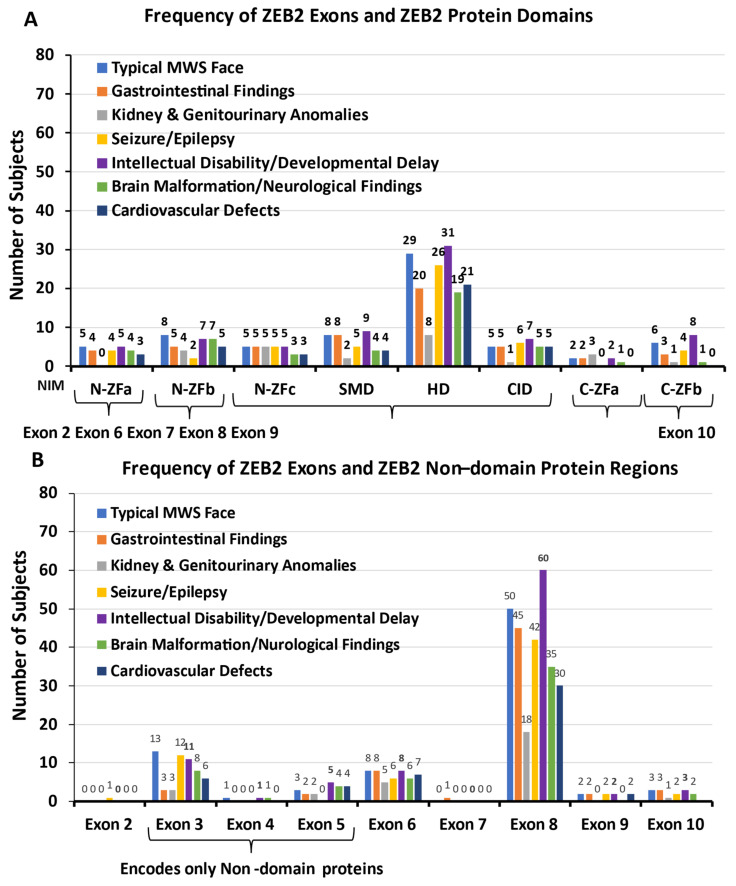
(**A**) ZEB2 exon variants with protein domains, clinical features and involvement of multiple organ systems. (**B**) ZEB2 exon variants with non-domain protein regions, clinical features and involvement of multiple organ systems. Exons 3, 4 and 5 encode only non-domain protein regions. Data utilized from literature sources [8,54,57,58,59,60,61,62,63,64,65,66,67,68,69,70,71,72,73].

The six ZEB2 protein domains and non-domain regions were evaluated in relationship to clinical features and organ systems (see Figure 6A,B). For example, ZEB2 protein domain involvement accounted for 46% of cases with a typical MWS face, as noted in Figure 6A, and non-domain protein regions were seen in 54% of cases, noted in Figure 6B. Among the protein domains, the most often affected was the HD domain, encoded by exon 8. Among the non-domain protein regions, 62% were encoded by exon 8 and 21% by exons 3, 4 and 5. ZEB2 protein domain involvement accounted for 45% of gastrointestinal issues, 45% of kidney and genitourinary issues, 44% of seizures/epilepsy, 45% of intellectual disability/developmental delay, 43% of brain malformations/neurological findings and 46% of cardiovascular defects (see Figure 6A). All organ systems showed the highest number of ZEB2 protein domains involved and exon 8 encoded the HD domain, then followed by the SMD domain. Only individuals with intellectual disability/developmental delay showed higher exon 10 disturbances. The non-domain protein regions accounted for 55% of gastrointestinal problems, 55% of kidney and genitourinary issues, 56% of seizures/epilepsy, 55% of intellectual disability/developmental delay, 57% of brain malformations/neurological findings and 54% of cardiovascular defects. The ZEB2 non-domain protein regions clustered into three regions, with the highest number recorded for exon 8, followed by exons 3 and 6, while exon 3 coded only non-domain regions (see Figure 6B). When comparing exon 8 mutations with non-exon 8 and organ systems, gastrointestinal findings (i.e., Hirschsprung and constipation) were significantly more involved (chi-square with Yates correction; *p* = 0.02). When comparing frameshift defects with no frameshift defects, the typical MWS face showed more defects in the non-domain protein regions (chi-square with Yates correction; *p* = 0.02).

## 4. Conclusions

Mowat–Wilson syndrome (MWS) is a complex and rare genetic disorder that commonly presents with developmental delays and intellectual disabilities, distinct facial dysmorphism, including a broad nasal bridge with a medial flair to the eyebrows, prominent deep-set eyes, a prominent chin and uplifted ear lobes with characteristic central depressions. Additionally, cases often exhibit congenital heart defects, gastrointestinal issues such as Hirschsprung disease, kidney and genital abnormalities, brain malformations, learning deficits and epilepsy. However, the severity and combination of features can vary, highlighting the genetic heterogeneity of the condition, as described in this report.

Mowat–Wilson syndrome results from pathogenic variants in the ZEB2 gene, which is located on chromosome 2q22 and plays a crucial role in embryonic development, influencing the formation and differentiation of multiple tissues and organs [36,44,74]. This gene is essential for various cellular processes required for proper development and function. Variants in the ZEB2 gene disrupt these processes, leading to the diverse clinical manifestations observed in MWS, varying based on the size, location and type of gene defect.

The ZEB2 protein plays a critical role in various organs and tissues before birth and in signaling pathways that regulate early growth and development. As a transcription factor, it binds to specific regions of DNA and regulates gene activity. The ZEB2 protein appears to be particularly important for the development of the neural crest, a group of cells in the early embryo that migrate to form portions of the nervous system, endocrine glands, pigment cells, smooth muscle and other tissues in the heart and many tissues in the face and skull, as reviewed by St. Peter et al. [8]. The ZEB2 protein is also active in cells that are not directly derived from the neural crest, but through growth factor regulation and timely repression impacting development of the digestive tract, skeletal muscles, kidneys and other organs [5,7,19,36]. The ZEB2 protein also regulates the epithelial–mesenchymal transition, a process essential for growth and development, wound healing and cancer metastasis. Disruptions in this process can have widespread effects on multiple organs and tissues, as seen in MWS.

The ZEB2 gene consists of ten exons that encode six protein domains and seven non-domain regions of variable size and function [8]. ZEB2 gene mutations often result in a loss of function of the ZEB2 protein, which leads to a range of developmental abnormalities. Clinical presentations can vary and while genetic testing can confirm the presence and type of ZEB2 gene mutation or variant, predicting the exact clinical outcome based on the location within the gene has been challenging [2,3,51,52,53,55,75]. In our review of 191 reported patients with MWS and ZEB2 gene defects, frameshift was present at 45% and nonsense at 38%. These were the most common mutations, followed by deletion at 6% and missense variants at 5%. Frameshift and nonsense variants and gene deletions are considered the most deleterious and, when detected, impact multiple organ systems’ development and function.

In our study, exon 8 encodes the majority of the ZEB2 protein, which is on average several-fold higher than for any of the remaining eight exons encoding the ZEB2 protein. Mutations or deletions in this exon disrupt the ZEB2 protein’s function, with frameshift and nonsense variants being the most common at contributing to a variety of developmental issues. Exon 8 is particularly important because of its functional significance, encoding all or part of four of the six functional protein domains widely expressed across multiple tissues, including in embryonic development. Consequently, mutations in exon 8 often result in pleiotropic effects, impacting multiple organs and systems [2,6,8,36]. This exon encodes about 60 percent of the ZEB2 protein domains and, not surprisingly, shows the highest number of associations, with involvement in nearly all organ systems, including the key clinical features seen in MWS. In our study, significantly more exon 8 mutations were found compared with other exons for gastrointestinal findings (i.e., Hirschsprung and constipation). In addition, significantly more frameshift defects were found in the non-domain protein regions, specifically for typical MWS face development, further indicating a role for non-domain regions having clinical significance.

ZEB2 gene mutations in non-domain protein regions may also contribute to MWS but are milder in clinical presentation, as described, or with different phenotypic findings. These regions may not directly affect the gene’s DNA-binding or transcriptional activity, but could impact protein stability, localization or secondary interactions and disturb biological pathways. Typical MWS facial features, developmental delay and intellectual disability were also represented in gene defects found in encoding non-domain protein regions that encompass exons 3, 4 and 5.

A better understanding of these non-domain regions may be important to clarify the full impact of ZEB2 gene mutations, particularly for MWS patients with atypical or less severe presentations as well as ZEB2 gene defects. In those with larger genetic defects such as chromosome deletions and truncation mutations, one would anticipate more severe clinical findings. Hence, one of the key challenges in understanding MWS is the lack of consistency in reporting, classifying and defining clinical features, which complicates efforts to establish more precise genotype–phenotype relationships in this rare disorder. Patients with Mowat–Wilson syndrome can present with a wide spectrum of phenotypic findings, with marked variability, complicated by a limited lifespan which restricts our ability to fully comprehend the natural history, long-term characteristics and outcomes with associated comorbidities. Additional studies are needed to gain more valuable insights into genotype–phenotype relationships and in the pathophysiology for rare genetic disorders, as explored in our report on MWS.

In summary, this review summarized the clinical features in MWS and explored genetic defects and phenotype relationships in affected individuals. This review sought to enhance our understanding of how specific ZEB2 gene variants influence clinical presentation in MWS by examining both published and unpublished databases collated previously by St. Peter et al. [8]. These insights are crucial for improving clinical surveillance after identifying the ZEB2 gene defect, guiding genetic counseling and developing targeted management strategies for individuals with this complex disorder. Given the broad range and severity of symptoms in MWS, treatment typically focuses on managing individual symptoms, with few evidence-based guidelines to inform care. This review addressed gaps in the literature by summarizing the documented genetic and phenotypic relationships found in MWS and informing a multi-system surveillance of patients diagnosed with specific genetic defects, as the phenotype can vary. Families, patients and physicians would greatly benefit from a deeper understanding of the genotype–phenotype relationship to explain clinical variability, develop healthcare guidelines and allow for the better monitoring of individuals with MWS throughout their lives and for natural history.

## Figures and Tables

**Table 1:** Types of ZEB2 gene defects related to clinical features and organ system involvement in Mowat–Wilson syndrome.

	Frameshift	Nonsense	Missense	Large Deletion	Small Deletion	Others
**Typical MWS face**	46% (n = 74)	38% (n = 62)	6% (n = 9)	2% (n = 4)	6% (n = 10)	2% (n = 3)
**Gastrointestinal findings**	50% (n = 63)	34% (n = 43)	6% (n = 7)	2% (n = 2)	6% (n = 8)	2% (n = 2)
**Kidney and genitourinary anomalies**	43% (n = 24)	39% (n = 22)	5% (n = 3)	2% (n = 1)	7% (n = 4)	4% (n = 2)
**Seizure/epilepsy**	43% (n = 55)	43% (n = 55)	4% (n = 5)	3% (n = 4)	5% (n = 6)	2% (n = 2)
**Intellectual disability/developmental delay**	46% (n = 83)	37% (n = 67)	6% (n = 10)	4% (n = 8)	6% (n = 10)	1% (n = 3)
**Seizure/epilepsy**	43% (n = 55)	43% (n = 55)	4% (n = 5)	3% (n = 4)	5% (n = 6)	2% (n = 2)
**Brain malformation/** **neurological findings**	50% (n = 60)	32% (n = 39)	3% (n = 4)	6% (n = 7)	7% (n = 9)	2% (n = 2)
**Cardiovascular defects**	46% (n = 49)	39% (n = 41)	1% (n = 1)	4% (n = 4)	8% (n = 9)	2% (n = 2)

## Data Availability

Data are contained within the article.
